# Neuroprotective Effect of Melatonin Loaded in Human Serum Albumin Nanoparticles Applied Subconjunctivally in a Retinal Degeneration Animal Model

**DOI:** 10.3390/pharmaceutics17010085

**Published:** 2025-01-10

**Authors:** Sofia Mickaela Martinez, Ayelen Inda, Maximiliano Nicolás Ríos, Carolina del Valle Bessone, Abril Bruera Bossio, Mario Eduardo Guido, José Domingo Luna Pinto, Daniel Alberto Allemandi, Daniela Alejandra Quinteros

**Affiliations:** 1 Unidad de Investigación y Desarrollo en Tecnología Farmacéutica (UNITEFA), CONICET and Departamento de Ciencias Farmacéuticas, Facultad de Ciencias Químicas, Universidad Nacional de Córdoba, Córdoba 5000, Argentina; sofia.martinez@unc.edu.ar (S.M.M.); ayelen.inda@unc.edu.ar (A.I.); cbessone@unc.edu.ar (C.d.V.B.); abril.bruera@mi.unc.edu.ar (A.B.B.); daniel.allemandi@unc.edu.ar (D.A.A.); 2 Centro de Investigaciones en Química Biológica de Córdoba (CIQUIBIC), Facultad de Ciencias Químicas, Universidad Nacional de Córdoba, Córdoba 5000, Argentina; maxi.rios@unc.edu.ar (M.N.R.); mario.guido@unc.edu.ar (M.E.G.); 3 Escuela de Ciencias de la Salud, Universidad Nacional de Villa Mercedes, Villa Mercedes 5730, Argentina; 4 Área de Cirugía Vítreo y Retina, Centro Privado de Ojos Romagosa S.A. y Fundación VER, Córdoba 5000, Argentina; fundacionver@gmail.com

**Keywords:** human serum albumin nanoparticles, melatonin, neurodegenerative eye diseases, neuroprotection

## Abstract

Background/Objectives: Neurodegenerative ocular diseases, such as age-related macular degeneration (AMD) and glaucoma, represent growing public health concerns. Oxidative stress plays a key role in their development, damaging retinal cells and accelerating disease progression. Melatonin (Mel) is a potent antioxidant with neuroprotective properties; however, it faces limitations such as low solubility. This study proposes the use of human serum albumin nanoparticles (Np-HSA) to enhance the delivery of Mel to the posterior segment of the eye and evaluates its neuroprotective and anti-apoptotic effects on the retina. Methods: A model of retinal degeneration was induced in New Zealand albino rabbits using cytotoxic and oxidative agents. Np-HSA-Mel nanoparticles were administered subconjunctivally, and cellular viability and retinal functionality were assessed using flow cytometry and pupillary light reflex (PLR). Histological and immunohistochemical studies, including the TUNEL assay, were performed to analyse cell survival and apoptotic index. Results: Np-HSA-Mel significantly preserved pupillary function and cell viability, demonstrating lower apoptosis compared to Mel solution and Np-HSA alone. Histologically, eyes treated with Np-HSA-Mel exhibited fewer structural alterations and greater cellular organisation. The TUNEL assay confirmed a significant reduction in the apoptotic index of retinal ganglion cells (RGCs) treated with Np-HSA-Mel. Conclusions: Np-HSA-Mel effectively overcame ocular barriers, achieving greater neuroprotective efficacy at the retinal level. These findings highlight the synergistic potential of albumin and Mel in treating neurodegenerative ocular diseases, opening new perspectives for future therapies.

## 1. Introduction

Neurodegenerative eye diseases such as age-related macular degeneration (AMD) and glaucoma are growing public health problems worldwide. Millions of people are affected by these conditions, which cause significant vision loss [1,2]. Oxidative stress is a key factor in the development of these diseases. This means that there is an imbalance in the eye cells where harmful substances called reactive oxygen and nitrogen species are produced. These substances damage retinal cells and accelerate the progression of diseases such as AMD and glaucoma [3,4]. These diseases not only affect people’s quality of life but also represent a major economic burden on healthcare systems. Oxidative stress is a silent enemy that contributes to vision loss in many people [2]. Understanding this process is fundamental for developing new treatments and preventing these diseases. A new model of retinal degeneration was developed by our research group in albino rabbits by injecting glutamate (GLUT) and L-buthionine-S, R-sulfoximine (BSO) into their eyes [5]. This combination induced oxidative stress, damaging retinal ganglion cells (RGCs), leading to cell death. This model replicates the mechanisms of retinal damage observed in ocular neurodegenerative diseases, facilitating the evaluation of new antioxidant agents that may protect RGCs from injury [5,6]. Melatonin (Mel) or N-acetyl-5-methoxytryptamine, an indolamine synthesised and secreted mainly by the pineal gland which influences circadian rhythms, seasonal behavioural changes, and acts as a free radical scavenger and antioxidant, is proposed as a therapeutic strategy to combat oxidative stress [7]. According to other studies, Mel has properties such as a high antioxidant effect and free radical scavenger, protecting the photoreceptor outer segment and other ocular cells from light-induced oxidative damage [8]. Studies show that Mel and its metabolites have direct and indirect antioxidant properties and can detoxify free radicals, thus preventing cell death and apoptosis; they can also protect intracellular lipids, proteins, and nucleic acids [8,9]. Several experimental models, including those based on diabetic retinopathy and glaucoma, have shown that the exogenous administration of Mel acts as a neuroprotective agent, preventing damage to the optic nerve and protecting RGCs from degeneration and cell death by apoptosis [10,11]. These combined actions make Mel an effective antioxidant and anti-apoptotic agent with widespread action, highly beneficial for the treatment of neurodegenerative ocular pathologies. However, its unfavourable physical and chemical characteristics, such as low solubility and low permeability at the corneal level, indicate that the topical ocular application has a poor pharmacological response at the posterior segment of the eye [12]. In this context, nanoparticles constitute a promising technological strategy as drug delivery systems, capable of overcoming ocular biological barriers and achieving pharmacological effects on the internal ocular structure [13]. This requirement is particularly critical in the context of advancing chronic neurodegenerative diseases, where the therapeutic effect relies exclusively on drug distribution and bioavailability within the internal ocular structures [13].

Nanoparticles such as niosomes, extracellular vesicles [14], and liposomes [15] protect melatonin from degradation and allow for controlled, sustained release. Melatonin-loaded Nps show superior antioxidant, anti-inflammatory, and anti-tumour properties compared to free melatonin. Applications include the treatment of various diseases such as cancer, heart disease, neurodegenerative diseases, and the promotion of tissue regeneration. However, in many cases, their clinical implementation is limited by challenges such as low yields, high production costs, and the need for functional optimisation for specific applications [14]. In this study, human serum albumin (HSA), the most abundant protein in human blood, was utilised. HSA is naturally derived and highly suitable for the production of nanoparticles (Nps) for drug delivery purposes. As a nanoparticle-forming material, this protein has several favourable properties: it is non-toxic [16], biodegradable [17], and lacks antigenic activity [18,19]. In addition, it can incorporate a significant amount of drugs into the cavities of the nanoparticles due to the different drug-binding sites present in the albumin molecule [20].

Human serum albumin nanoparticles (Np-HSA) developed using the thermal stabilisation method represent a promising strategy for the treatment of ocular pathologies. This method is efficient, scalable, low cost, and avoids the use of toxic agents such as glutaraldehyde. Additionally, it demonstrates strong biopharmaceutical properties, particularly in sustained drug release and physical stability, both in suspension and in reconstitutable solid form, facilitating transport and storage. Finally, it is suitable for various administration routes, such as topical and subconjunctival routes, without causing significant irritation in in vivo tests [21].

The aim of our study was to evaluate the neuroprotective and anti-apoptotic effects of Mel in the Np-HSA systems (Np-HSA-Mel) on retinal cells, mainly in RGCs. To evaluate the effect of Mel, an in vivo assay was performed in which Np-HSA-Mel was administered subconjunctivally into RDM induced by oxidative and cytotoxic agents. Changes in retinal cell viability and functionality were assessed by flow cytometry and pupillary light reflex (PLR) as indicators of neuro-ophthalmic disorder. Specific ex vivo analyses were used to evaluate nanoparticle permeation through the sclera and cornea. The performance of RGCs was also evaluated, including histological studies and immunohistochemical assays, to assess cell survival and the incidence of apoptosis.

## 2. Materials

HSA was isolated from UNC™ human serum albumin (20% *w*/*v* human serum albumin, Laboratory of Hemoderivatives, UNC, Córdoba, Argentina). This product contains excipients such as sodium caprylate sodic and N-acetyl tryptophan (NaT), both at a concentration of 0.08 mmol/g of albumin, with the main purpose of stabilising the protein against temperature changes [22]. The frozen alcohol precipitation method was used for purification. This method removed the excipient sodium caprylate, while the excipient N-Acetyl tryptophan was reduced by 86.2% according to HPLC analysis [23], obtaining HSA with 99.7% purity. Melatonin was acquired from Parafarm (Buenos Aires, Argentina). Glutamate, L-butionine-S, R-sulfoximine, proteinase K, hydrogen peroxide, propidium iodide and formaldehyde, papain, and an in situ cell death detection kit (TUNEL, “terminal deoxynucleotidyl transferase-mediated dUTP nick end labelling”) were purchased from Sigma-Aldrich (St. Louis, MO, USA). Hematoxylin and eosin were acquired from Biopack (Buenos Aires, Argentina). Calcein Green was purchased from Invitrogen™ (Thermo Fisher Scientific, Carlsbad, CA, USA). Proparacaine hydrochloride 0.5% was purchased from Anestalcon^®^ Alcon Laboratory (Buenos Aires, Argentina).

## 3. Animals

Rabbits (2–2.5 kg) were housed in individual enclosures with free access to food and water. They were maintained on a 12/12 h light/dark cycle. All experiments were conducted following the guidelines set forth by the Association for Research in Vision and Ophthalmology (ARVO) for the use of animals in research, the Directive of the Council of the European Communities (86/609/EEC), and the Institutional Care and Use Committee (CICUAL) of the Faculty of Medicine, National University of Córdoba (Res. CE 2021-00338577-UNC-SCT-FCM). Every effort was made to minimise the number of animals used in each experiment.

## 4. Methods

### 4.1. Preparation of Melatonin Formulations

The nanoparticles of HSA, both loaded with (Np-HSA-Mel) and without (Np-HSA) melatonin, were prepared using a combined desolvation and thermal stabilisation method, as described by Martinez et al. (2022) [21]. Np-HSA-Mel was formulated at a concentration of 1 mg/mL of Mel, and a Mel solution (Mel Sol) at the same concentration was used as a control. The Mel solution was prepared in a physiological medium under isosmotic conditions with a pH of 7.2.

### 4.2. Physicochemical Characterisation

#### 4.2.1. pH

The pH of the formulations was determined using a potentiometer (SevenMulti, Mettler Toledo, Columbus, OH, USA). Each sample was analysed in triplicate (*n* = 3).

#### 4.2.2. Particle Size, Polydispersity Index and Zeta Potential

Dynamic light scattering (DLS) equipment (Malvern Zetasizer 3600, Malvern, UK) was used to determine the particle size, polydispersity index (PDI), and electrokinetic potential (ZP) after the dilution of Np-HSA and Np-HSA-Mel samples with ultrapure water (1:500). Each sample was analysed in triplicate (*n* = 3) at 25 °C.

#### 4.2.3. Encapsulation Efficiency (EE%) of Mel

The quantification of Mel present in Np was carried out using high-performance liquid chromatography (HPLC) Agilent^®^ Series 1100 (Agilent, Santa Clara, CA, USA), with a Phenomenex^®^ C18 column (Phenomenex, Torrance, LA, USA). An acetonitrile/water solution (65:35 *v*/*v*) was used as the mobile phase. The flow rate was set at 0.9 mL/min at 30 °C using UV detection at 220 nm, and the injection volume was 20 µL [24]. Np samples were cleaved with NaOH and filtered (0.45 µm) before HPLC quantification.

The encapsulation efficiency (*EE*) percentage was calculated using the following equation:

*EE* (%) = (encapsulated *Mel* (mg)/*total* Mel (mg)) × 100 (1)

#### 4.2.4. Determination of Remaining NaT in Np-HSA

To determine the amount of residual NaT in the purified HSA, an HPLC technique developed for HSA quantification was used. Chromatographic runs were performed on a C18 column (150 × 4.60 mm; 5 μm particles) with a mobile phase consisting of ACN:H_2_O:H_3_PO_4_ (50:50:0.1 *v*/*v*/*v*) with 0.1% triethanolamine (TEA) at a flow rate of 0.8 mL/min and a detection wavelength of 220 nm. The retention time of NaT was 2.25 min [23].

A calibration curve was constructed using serial dilutions of standard NaT in a concentration range of 0.00125 to 0.1 mg/mL. The curve was performed in duplicate, achieving a close fit to a straight line, as evidenced by a correlation coefficient (R) of 0.9999.

For this, the non-encapsulated NaT in the supernatants was quantified, and the amount of NaT inside the nanoparticles was indirectly calculated by subtracting the amount found in the supernatants from the total amount of the excipient remaining from the purification process associated with HSA.

### 4.3. Ex Vivo Transcorneal and Transscleral Permeation Assay of Np-HSA-Mel

Horizontal bicompartmental cells were used to perform ex vivo assays using isolated rabbit corneas and sclera located between the two compartments. In the case of the cornea, it was incorporated with the corneal endothelium in the direction of the receptor solution (PBS pH = 7.4) in a volume of 4 mL, while the epithelial side was kept in contact with the donor medium containing the formulation to be tested. The sclera was placed with the lamina fusca in the direction of the receptor solution and the episclera was in contact with the evaluated formulation. The temperature in the diffusion chamber was kept constant (36.0 ± 0.5); to obtain a homogeneous mixing of the solutions and maintain tissue viability, an O_2_-CO_2_ mixture (95:5) was passed through both compartments during the experiment.

To remove the corneas and sclerae, the animals were anesthetised with a mixture of 0.75 mL/kg of ketamine and 0.25 mL/kg of xylazine. Subsequently, the animals were sacrificed with a mixture of 10% O_2_ and 90% CO_2_ in a sealed acrylic chamber. Corneas were removed with a 2 mm scleral remnant, while sclerae were removed in 1.2 cm^2^ fragments.

Samples were removed from the receptor chamber at 15, 30, 45, 60, 75, 90, 105, and 120 min and immediately replaced with the same volume of previously tempered medium. The permeated drug concentration was then quantified by UV–Vis spectroscopy at 279 nm. Assays were performed in duplicate, and melatonin sink conditions were maintained.

A linear regression analysis of the obtained diffusion data allowed calculating the following parameters:

- Permeation rate (v): ΔQ/Δt (µg/min), where Q is the amount of Mel diffused through the cornea or sclera at time t.
- Steady-state flux (J): v/A (µg/min·cm^2^), where A is the effective available tissue surface area.
- Apparent permeability coefficient (P_app_): J/Ci, where Ci is the initial drug concentration of the donor medium.

### 4.4. Application of Np Formulations in a Retinal Degeneration Model (RDM) in New Zealand Rabbits

The in vivo experiments were conducted using an RDM for animals, by the intravitreal administration of a single dose of GLUT and BSO to both eyes of New Zealand rabbits, as described by Bessone et al. 2019 [5]. Three formulations were applied subconjunctivally under the experimental conditions: Np-HSA, Np-HSA-Mel, and Mel Sol at a Mel concentration of 1 mg/mL. Control eyes damaged with RDM (designated as RDM) and untreated eyes (designated as HEALTHY) were used. Subconjunctival applications were performed at the upper bulbar conjunctiva in a volume of 100 μL, using 0.5 mL tuberculin syringes with an incorporated 30 G needle. After subconjunctival injections, 4% iodopovidone solution was applied topically as prophylaxis. The RDM was spread over nine days, during which time all of the following tests were performed.

### 4.5. Evaluation of the Neuroprotective Effect on Total Retinal Cells

#### 4.5.1. Pupillometry

The pupillary light reflex (PLR) is used to assess retinal functionality in neuro-ophthalmic disorders [25,26]. The animals were kept in the dark for 12 h before starting the study and were adapted to a dim red-light environment (1 lux) for 10 min. The rabbits were held and restrained so that the cornea and camera lens were 6 cm apart in a parallel plane. The eye of each animal was photographed using a smartphone camera (Samsung, 50 megapixels, 3x magnification) for 30 s after exposure to white light (350 lux), and serial images were extracted every second. Linear pupil constriction was measured from the images acquired from the recordings using ImageJ image analysis software. Data were normalised to the maximum pupil diameter immediately after light onset.

The visual function of healthy eyes was first compared to that of RDM; the effectiveness of treatments to restore pupil constriction was then assessed. The tests used for Mel Sol *n* = 3 eyes, Np-HSA-Mel *n* = 4 eyes and HEALTHY, RDM, and Np-HSA *n* = 5 eyes.

#### 4.5.2. Retinal Cell Viability Using Flow Cytometry

Flow cytometry was used to assess retinal cell viability, following the methodology previously described by Rios et al. (2023) [27]. Briefly, to compare healthy eyes with RDM-treated eyes and assess the effect of Mel Sol, Np-HSA and Np-HSA-Mel formulations on cell viability in RDM, eyes were enucleated on day 9. Retinas were digested with 4% papain for 15 min at 37 °C, followed by centrifugation at 300× *g* for 10 min. The papain was then removed, and the samples were resuspended in 1X PBS. The samples were incubated with 50 μM calcein green-AM, a live-cell dye, for 30 min according to the manufacturer’s protocol (Invitrogen^®^), and 50 μg/mL propidium iodide was added to differentiate dead cells. All samples were processed by flow cytometry using a FACS Canto II instrument (BD Biosciences, Milpitas, CA, USA) with a minimum of 100,000 cells per sample. Each sample was analysed in triplicate. Data analysis was performed using FlowJo software 10.0.7.r2 (Verity Software House, Topsham, ME, USA). Cell viability was determined based on the numerical frequency of calcein-positive and propidium iodide-positive cells plotted on a histogram.

### 4.6. Evaluation of Neuroprotective Effect in Retinal Ganglion Cells

#### 4.6.1. Histopathological Studies

Nine days after RDM exposure, the animals were sacrificed, and their globes were enucleated. The eyes were fixed in a 4% paraformaldehyde solution in PBS (pH 7.2) at room temperature. The eyes were dissected (10 μm) in the horizontal plane of the optic nerve and stained with haematoxylin and eosin for analysis using optical microscopy (Olympus^®^ BX41 microscope, Tokyo, Japan; Infinity 1^®^ camera) [11,28]. The viability of RGCs was assessed by analysing retinal integrity, including the optic nerve. To do this, retinal sections were organised into sets with an equal number of images for all treatments to be evaluated, and manual RGC counts were performed using ImageJ software as support. This study was conducted in triplicate (*n* = 3 eyes) for each treatment (RDM, Sol Mel, Np-has, and Np-HSA-Mel).

#### 4.6.2. Detection of Apoptosis In Situ Using the TUNEL Assay

Apoptotic cells were visualised using the TUNEL assay according to the supplier’s protocol. Briefly, the tissue samples were incubated with 20 µg/mL proteinase K for 15 min. Subsequently, they were rinsed with distilled water and incubated with a 3% hydrogen peroxide (H_2_O_2_) solution in PBS for 5 min at room temperature to prevent enzymatic activity. After rinsing the slides with PBS, the samples were placed inside a humidified chamber in the dark and incubated with the TUNEL reaction mixture at 37 °C for 60 min to allow the reaction to occur. A colour reaction was initiated by the addition of diaminobenzidine (DAB), and the sections were counterstained with 0.5% methyl green before analysis by light microscopy (Olympus BX41). By light microscopy, the apoptotic nuclei appeared brown and were classified as positive, whereas the non-apoptotic cells were stained blue. TUNEL-positive cells were quantified relative to the total cell count in the RGC layer and expressed as a percentage of the apoptotic index (AI%). The analysis was performed digitally using ImageJ, with images captured at 20x magnification. This study was replicated three times per treatment (RDM, Mel Sol, Np-HSA, and Np-HSA-Mel).

### 4.7. Statistical Analysis

Statistical studies included one-way analysis of variance (ANOVA) with Bonferroni or Kruskall–Wallis post hoc tests for non-parametric analysis (significance at *p* < 0.05 or *p* < 0.1) or T-test using the Mann–Whitney test, as appropriate. All graphs and statistical tests were performed with GraphPad Prism 8.

## 5. Results and Discussion

### 5.1. Physicochemical Characterisation of Formulations

Results from previous studies conducted by this lab have led us to conclude that Np-HSA-Mel has high potential as an ophthalmic drug delivery system, releasing melatonin over a prolonged period and with almost no toxic effects on ocular tissues [21].

Using the combined technique of desolvation and thermal stabilisation, Np-HSA and Np-HSA-Mel systems were obtained, whose main physicochemical characteristics are detailed in Table 1. Regarding particle size, measurements showed a hydrodynamic radius between 160 and 170 nm, with a standard deviation of less than 5% in both cases, suggesting high homogeneity in particle size. This was corroborated by analysing the PDI values (0.06), which showed a very narrow size distribution, indicating that it is almost monodisperse system [29]. The pH of the dispersions ranged between 7.3 and 7.4, close to a physiological pH. The obtained ZP values were approximately −30 mV in deionized water at the pH levels indicated in the table, suggesting high stability with minimal aggregation [30]. This can be explained by the increase in the Np surface charge as the pH moves away from the isoelectric point of HSA (pH 5.0) [31,32]. The EE % of Mel in the system was relatively low at 21.3%, and attributed to its poor solubility and weak interaction with HSA. However, the developed production method enables the freeze-drying and reconstitution of the formulation at the desired melatonin concentration (1 mg/mL) while preserving its physicochemical properties, unchanged [21]. Finally, the remaining NaT content in the systems ranged between 0.06 and 0.09 mg/mL.

### 5.2. Ex Vivo Assays: Transcorneal and Transscleral Permeation Studies

The intravitreal route is highly effective in achieving high drug concentrations in the eye, but it is invasive and can cause serious complications such as bleeding or infection [33]. For this reason, alternative routes are being sought, such as the topical route, which is the most common and accepted by patients. However, less than 5% of drugs manage to penetrate the corneal barrier and reach the aqueous humour, which requires frequent dosing to maintain adequate therapeutic levels and fails to achieve pharmacologically active concentrations in the tissues of the posterior segment [34]. The subconjunctival route is less invasive than intravitreal and more effective than topical in reaching the posterior part of the eye. However, it can cause temporary discomfort and does not always reach the retina at the desired concentrations [35]. Therefore, these alternative routes of administration to reach the posterior segment of the eye were explored.

Studies indicate that, although the cornea could be considered the primary route for drug permeation after topical application, larger molecules predominantly enter the eye through diffusion across the sclera. While the cornea is relatively impermeable to molecules larger than 1 kDa, dextran (40 kDa) and serum albumin (69 kDa) can easily penetrate human scleral tissue [34].

Although the corneal surface is more permeable than the stratum corneum, it is relatively impermeable compared to other epithelial tissues because of its tight cellular junctions [36]. Some studies postulate that scleral permeability may be up to 10 times greater than corneal permeability and is directly proportional to scleral thickness and total surface area [34].

**Figure 1:**
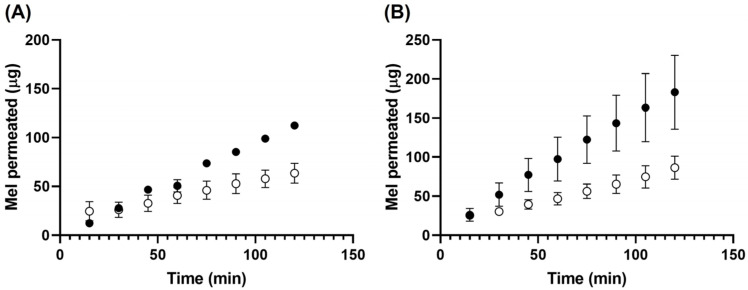
Permeation profiles of Mel in PBS (pH = 7.4) at 36 °C in cornea (**A**) and sclera (**B**) for Np-HSA-Mel formulation (white dots) compared to Sol Mel 1 mg/mL as a control (black dots).

Figure 1 displays the results of the transcorneal (Figure 1A) and transscleral (Figure 1B) permeation tests, demonstrating that Np-HSA-Mel exhibits a lower amount of active compound permeation through the cornea and sclera than that of the Mel solution used as a control. Additionally, it can be observed that Mel showed a higher percentage of permeation through the sclera than through the cornea.

Mel from Np-HSA-Mel passes through the cornea and sclera almost 2.5 times slower than from the Mel solution because Mel is released from the carrier system in a prolonged manner, as reported by Martinez et al. (2022) [21]. This is evidenced by the permeability coefficient Paap, with values of 1.21 × 10^−3^ and 1.89 × 10^−3^ for the Mel solution in the cornea and sclera, respectively, and 0.50 × 10^−3^ and 0.74 × 10^−3^ for Mel from Np-HSA-Mel in the cornea and sclera, respectively (Table 2).

These results are consistent with those reported by Netweera et al. who developed Mel-loaded niosomes, which demonstrated a lower and sustained permeation rate through the skin, whereas the control Mel gel passed freely into the receptor compartment [37].

### 5.3. In Vivo Assays: Evaluation of the Neuroprotective Effect of Mel from Np-HSA-Mel in RDM

#### 5.3.1. Subconjunctival Administration of Formulations

As previously described, both the Mel solution and Np-HSA-Mel showed greater permeability in the sclera than in the cornea. Additionally, research indicates that Np-HSA-Mel remained in the sclera for up to 7 days after injection, making it an effective depot system for sustained drug release.

#### 5.3.2. Evaluation Formulations of Retinal Cell Functionality

**Figure 2:**
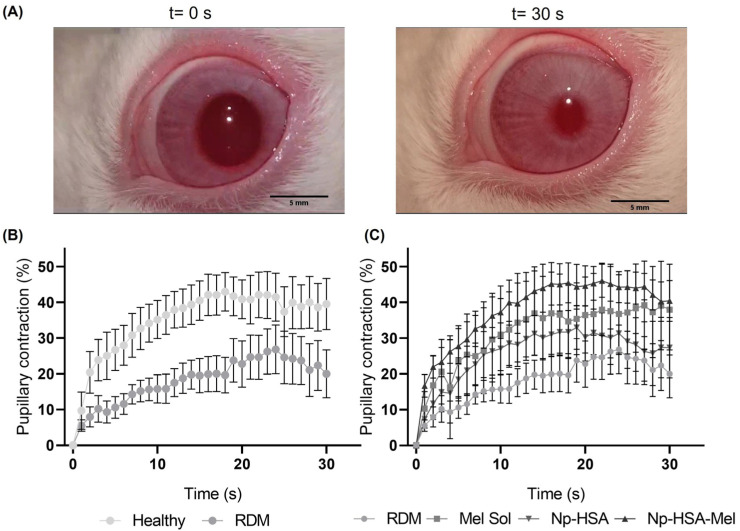
Pupillometry assay. (**A**) Images captured at 0 and 30 s after exposure to white light (350 lux), showing pupillary contraction. Analysis was performed using ImageJ software 1.52a. Scale bar: 5 mm. (**B**) Graph of pupillary contraction over time with white light (350 lux) for healthy eyes vs. eyes exposed to RDM. (**C**) Comparison between eyes exposed to RDM and treated with Mel Sol, Np-HSA, and Np-HSA-Mel. Results are presented as the mean ± standard error (*n* = 5 eyes/group).

The pupillary light reflex (PLR) assay can be considered a direct method because it observes the functionality of the pathway in real time. The PLR assesses the speed and magnitude of pupil constriction or dilation in response to light stimulation (Figure 2A). The use of chromatic pupillometry methods in patients with retinal or optic nerve diseases has demonstrated effectiveness in evaluating damage to both photoreceptors and intrinsically photosensitive retinal ganglion cells (ipRGCs) [38]. The objective of this study was to evaluate an RDM with specific apoptosis in RGCs [5] affecting PLR and to determine the impact of various formulations on retinal degeneration, as observed through PLR measurements.

As can be seen in Figure 2B, eyes subjected to RDM showed a decrease in pupil contraction with a maximum of (27.00 ± 6.86)% at 24 s compared to healthy eyes, which showed a maximum contraction of (40.04 ± 3.32)% at 18 s. On the other hand, as shown in Figure 2C, the maximum pupil contraction values in eyes treated with RDM followed by treatment were (39.15 ± 12.36)% at 27 s for Mel Sol, (33.64 ± 6.62)% at 22 s for Np-HSA, and (46.05 ± 4.64)% at 22 s for Np-HSA-Mel.

**Figure 3:**
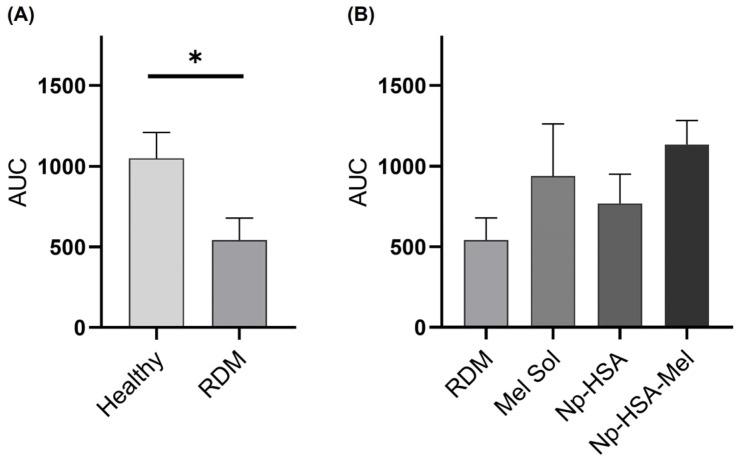
Cumulative AUCs for (**A**) healthy and RDM, and (**B**) RDM, Mel Sol, Np-HSA, and Np-HSA-Mel eyes with their respective standard errors (SEM) used for statistical analysis. Illumination was with white light (350 lux). Significant differences are observed in the *t*-test between AUCs for RDM-exposed eyes and healthy eyes (* *p* < 0.05; *n* = 5), as well as with Np-HSA-Mel treatment (*p* < 0.1) (non-parametric statistics, Kruskal–Wallis test; *n* = 5 eyes/group).

For the statistical analysis, the areas under the curve (AUC) were calculated for both plots and are presented in Figure 3A. Subsequently, statistical analysis was performed using the *t*-test; it was determined that there were statistically significant differences (*p* = 0.0419) between the cumulative AUC of healthy and RDM-exposed eyes. These results suggest that the RDM used can cause damage to the retina in the cells responsible for pupil contraction.

In the calculation of AUC for these treatments (Figure 3B), results indicated a notable tendency of Np-HSA-Mel to maintain pupillary contraction function compared to eyes subjected to RDM, with statistical differences observed via the Kruskal–Wallis test (α = 0.1, *p* = 0.06). In contrast, no significant differences were observed in the other formulations.

#### 5.3.3. Retinal Cell Viability by Flow Cytometry

**Figure 4:**
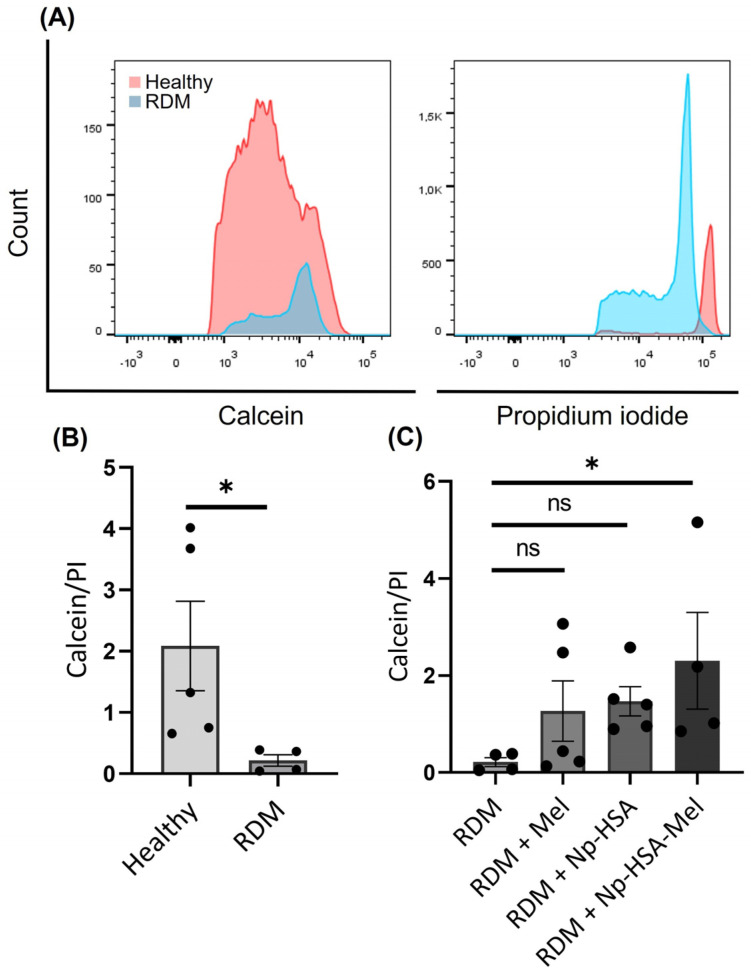
(**A**) Representative fluorescence histograms for calcein and PI channels show the comparison between healthy (pink) and RDM (blue) for the population tested. (**B**) Graph of the ratio between calcein-positive and PI-positive cells in healthy and RDM-treated eyes. A significant increase in the live cell count is observed in healthy eyes compared to RDM-exposed eyes (* *p* < 0.05) using the Mann–Whitney test (*n* = 5). (**C**) Graph of the ratio between calcein-positive and PI-positive cells for the eyes examined having their RDM-exposed eyes treated with Mel Sol, Np-HSA, and Np-HSA-Mel. Although an increasing trend was observed for all treatments, only Np-HSA-Mel showed statistically significant differences compared to RDM (* *p* < 0.05) using the Kruskal–Wallis test. Plotting the calcein/propidium iodide ratio (Figure 4B) for the population examined shows a quantitative and statistically significant increase in apoptosis induced by RDM compared to a healthy control (Mann–Whitney test, *p* < 0.05).

Initially, the ability of RDM to induce apoptosis in total retinal cells was evaluated and compared to that of a healthy control eye. As shown in the histograms (Figure 4A), calcein levels decreased in the RDM eye. In contrast, the histograms of propidium iodide-positive cells reveal a notable increase in RDM. These results indicate that RDM is sufficiently aggressive to reduce cell viability in total retinal cells.

The neuroprotective effect of Mel Sol, Np-HSA, and Np-HSA-Mel treatments on RDM-treated eyes was then assessed by quantifying total retinal cell viability (Figure 4C). Although there is a clear trend towards increased cell viability in all cases, only Np-HSA-Mel showed statistically significant differences (non-parametric statistics, Kruskal–Wallis test, *p* < 0.05) compared to RDM, indicating that this kind of treatment may have a neuroprotective effect at the retinal level. In addition, Np-HSA shows a strong trend towards increased cell viability in the population studied.

**Figure 5:**
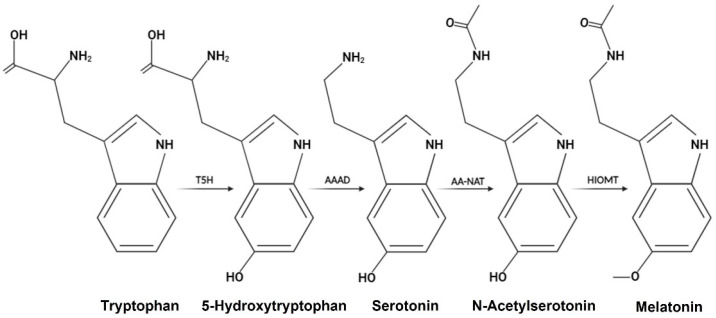
Representative diagram of melatonin synthesis.

In this context, several studies suggest that albumin treatment has neuroprotective effects, ameliorating neurobehavioral deficits, reducing oxidative stress, and promoting neuronal survival after intracerebral haemorrhage [39]. It has also been shown that the prolonged oral administration of albumin induces neuroprotective effects against cerebral ischaemia [40]. Additionally, some studies have reported that HSA protects glutamatergic neurons against oxidative stress-induced cell death [41] by increasing intracellular glutathione levels, which plays a crucial role in oxidative stress tolerance and cell survival [42]. Overall, these findings suggest that albumin has neuroprotective effects and could have therapeutic applications in the treatment of neurological disorders and neurodegenerative diseases. Note that the albumin used contains a residual stabiliser, N-Acetyl-Tryptophan (NAT), a precursor in Mel synthesis. First, tryptophan hydroxylase catalyses the conversion of tryptophan to 5-hydroxytryptophan (5-HTP). Next, L-aromatic amino acid decarboxylase (AAAD) converts 5-HTP into serotonin. In the rate-limiting step of synthesis, arylalkylamine N-acetyltransferase (AANAT) uses serotonin or 5-hydroxytryptamine to produce N-acetylserotonin (NAS). Finally, this compound is used by the final enzyme in the Mel synthesis pathway, N-acetylserotonin O-methyltransferase (HIOMT), which methylates NAS to convert it to Mel (Figure 5).

Various studies suggest that NAT has neuroprotective effects. For instance, Kumar et al. demonstrated that pre-treatment with NAT provided significant radioprotection in neuronal cells, reducing cell death and DNA damage induced by radiation [43]. Fernandes et al. assessed the effect of NAT on spatial memory impairments induced by aluminium in rats and found that NAT improved memory alterations, possibly by blocking substance P-mediated neuroinflammation and reducing oxidative stress [44]. These results suggest that there could be a synergy between the residual amino acid and Mel, along with the previously described properties of HSA.

### 5.4. Evaluation of Neuroprotective Effect at the Level of Retinal Ganglion Cells

#### 5.4.1. Cell Viability Assessment: Histological Assays

**Figure 6:**
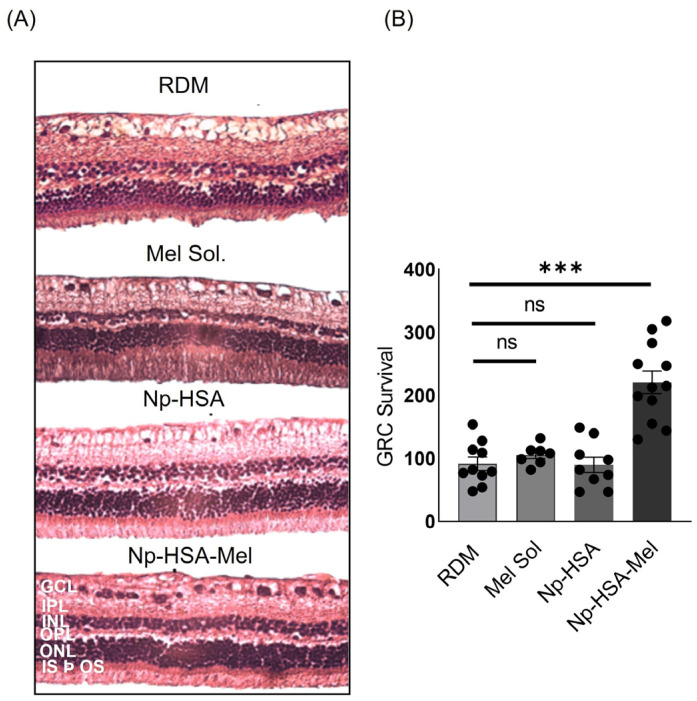
Cell viability of RGCs at 9 days post-MDR induction and subconjunctival injection of different treatments. (**A**) On the left, representative images of retinal sections stained with haematoxylin and eosin are shown, obtained by optical microscopy on the ninth days after MDR application and treatment; Mel solution (1 mg/mL), Np-HSAand Np-HSA-Mel (1 mg/mL). Structural and cellular differences in the various layers of the retina can be observed. The retinal layers are defined as follows: GCL: ganglion cell layer, IPL: inner plexiform layer; INL: inner nuclear layer; OPL: outer plexiform layer; ONL: outer nuclear layer; and IS þ OS: inner and outer photoreceptor segment. (**B**) On the right, the graph represents the quantitative analysis of the number of RGCs surviving in retinal tissues treated with the aforementioned formulations compared to the control. Data are presented as (mean ± SEM) for all groups (*n* = 3) obtained on the ninth day of treatment. Significant differences obtained by the Kruskal–Wallis test are indicated relative to the control value (*** *p* < 0.001).

As previously mentioned, RDM significantly induced apoptosis in RGCs. To evaluate the neuroprotective effects of Mel through different treatments, histological assays were conducted to determine cell viability in the RGC layer. Histological sections from eyes exposed to RDM and subsequently treated with Np-HSA-Mel showed fewer structural alterations and greater organisation and definition of cellular layers than those of control formulations (Figure 6A). Additionally, a higher number of surviving RGCs was observed in comparison to the RDM control (non-parametric statistics. Kruskal–Wallis test * *p* < 0.05). However, this effect was not observed in treatments with Mel Sol and Np-HSA, where histological sections demonstrated lower RGC viability without significant statistical difference as compared to the control (Figure 6B).

#### 5.4.2. Apoptotic Index Assessment: TUNEL Technique

**Figure 7:**
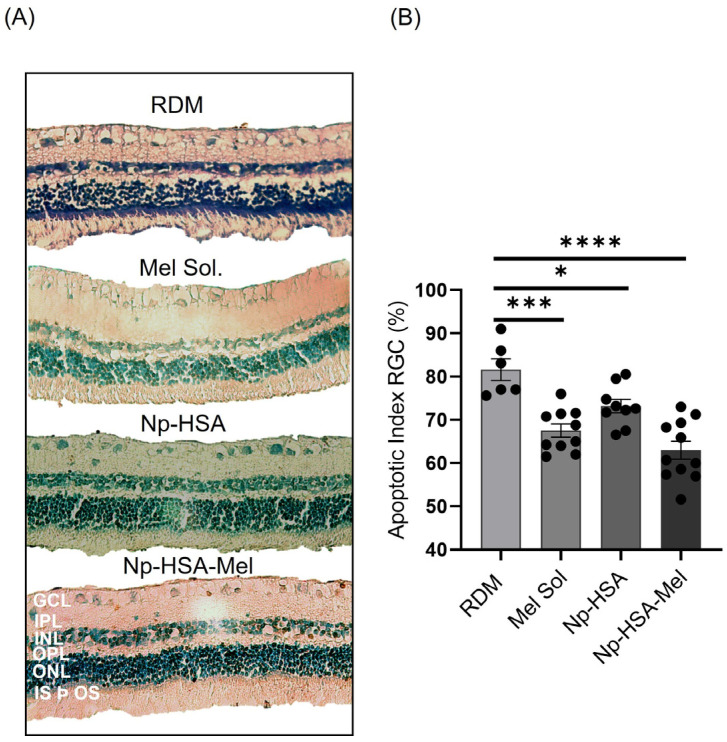
(**A**) Retinal images are representative of TUNEL assay for detecting cell death by apoptosis in RGCs after 9 days of MDR induction and treatment with the following: Mel solution (1 mg/mL), Np-HSA, and Np-HSA-Mel (1 mg/mL). Scale bar: 100 µm. (**B**) The bar graph presents the quantitative analysis of the proportion of TUNEL-positive RGCs for each treatment evaluated relative to the control. Results are shown as (mean ± SEM) for all groups (*n* = 3). Significant differences obtained by ANOVA are indicated in relation to control values (* *p* < 0.05; *** *p* < 0.001; **** *p* < 0.0001).

Based on the previous results, and to determine the percentage of retinal RGCs in an apoptotic state, an in situ immunohistochemical detection test was performed on retinal tissues using the TUNEL technique. For RDM, a significant increase in the apoptotic index (AI) was observed, with values of (81.63 ± 2.50)%, indicating that RDM induces RGC apoptosis. Additionally, it was found that treatments with Np-HSA (73.20 ± 1.56)%, Mel Sol (67.5 ± 1.53)%, and Np-HSA-Mel (63.0 ± 2.08)% showed a statistically significant decrease compared to the damage model (one-way ANOVA, *p* < 0.05) (Figure 7B). These results are consistent with those reported by Bessone et al. (2019) [5]. As in the findings of flow cytometry studies, Np-HSA showed an effect on neuroprotection in the RGC layer, possibly exerting a synergistic effect with Mel to enhance its efficacy.

## 6. Conclusions

In conclusion, the results obtained provide a comprehensive view of the neuroprotective effect of Np-HSA-Mel, Sol Mel, and Np-HSA at the level of the entire retina and the ganglion cell layer when administered subconjunctivally after the induction of RDM in New Zealand albino rabbits. The combination of techniques such as pupillometry and flow cytometry allowed for the assessment of retinal neuronal cells, while histological and immunohistochemical studies, such as the TUNEL technique, enabled the evaluation of cell viability and the apoptotic index in retinal ganglion cells.

The RDM-induced damage in the retina caused a significant decrease in pupil contraction, indicating damage to the cells responsible for the PLR. This pupillary impairment was associated with the presence of ocular abnormalities that can be extrapolated to pathologies such as glaucoma, macular degeneration, and diabetic retinopathy. These results were supported by flow cytometry, which showed that RDM had a significant effect on the reduction in total retinal cell viability. Among the treatments, Np-HSA-Mel was the most effective in preserving pupillary function and reducing cell apoptosis, both in the total cell population and in specific subpopulations. Np-HSA and Sol Mel also showed protective effects, although to a lesser extent than Np-HSA-Mel.

Histological studies showed fewer structural changes and greater cellular organisation in eyes treated with Np-HSA-Mel compared to RDM. A significant increase in retinal ganglion cell survival was also observed with Np-HSA-Mel (*** *p* < 0.001) in contrast to the retinal damage model. The TUNEL technique showed a statistically significant decrease in the apoptotic index of retinal ganglion cells with Np-HSA-Mel compared to RDM (**** *p* < 0.0001), suggesting a positive effect on cellular neuroprotection.

It is important to highlight that the use of nanoparticles allowed for the better delivery of Mel to the posterior segment of the eye, overcoming ocular barriers and enabling the greater efficacy of Np-HSA-Mel nanoparticles as a neuroprotective agent for retinal cells, primarily at the RGC level, in eyes that underwent RDM with Glu-BSO. The neuroprotective potential of the albumin present in the nanoparticles was also emphasised, allowing for a synergistic effect with Mel, resulting in greater therapeutic efficacy. This finding opens new perspectives in the treatment of neurological disorders and neurodegenerative diseases.

## Figures and Tables

**Table 1:** Main physicochemical characteristics of Np-HSA and Np-HSA-Mel systems indicating pH, size (nm), PDI, ZP, Mel EE (%), and NaT concentration (mg/mL) for each type of Np.

Formulation	pH	Size (nm)	PDI	ZP	Mel EE (%)	NaT Concentration (mg/mL)
Np-HSA	7.38 ± 0.06	168.3 ± 2.2	0.06 ± 0.03	−33.7 ± 0.5	-	0.058 ± 0.026
Np-HSA-Mel	7.32 ± 0.12	162.8 ± 4.9	0.06 ± 0.01	−32.8 ± 0.8	21.3 ± 1.9	0.093 ± 0.012

**Table 2:** Transcorneal and transscleral permeability parameters for Np-HSA-Mel and Mel solution. Values are presented as mean ± SD.

	Velocity (µg/min)	Flux (J) (µg/min·cm^2^)	Papp (×10^−3^)	µg Permeated (2 h)
Cornea				
Mel Solution	0.95 ± 0.02	1.21 ± 0.03	1.21 ± 0.03	112.45 ± 0.04
Np-HSA-Mel	0.39 ± 0.01	0.50 ± 0.01	0.50 ± 0.01	63.41 ± 10.25
Sclera				
Mel Solution	1.49 ± 0.37	1.90 ± 0.47	1.89 ± 0.47	183.01 ± 47.29
Np-HSA-Mel	0.58 ± 0.13	0.74 ± 0.17	0.74 ± 0.17	86.30 ± 14.90

## Data Availability

Data will be made available on request.
